# Effect of compounds on the purification and antibody preparation of the extracellular domain fragment of the receptor CD163

**DOI:** 10.1186/1743-422X-8-144

**Published:** 2011-03-29

**Authors:** Zong-Xi Cao, Fu-Rong Zhao, Kun Jia, Wei-Wei Sun, Ming-Fei Yan, Si-Hu Guo, Pei-Rong Jiao, Wen-Bao Qi, Gui-Hong Zhang

**Affiliations:** 1 Key laboratory of Animal Disease Control and Prevention, Ministry of Agriculture, College of Veterinary Medicine, South China Agricultural University, Guangzhou 510642, China

**Keywords:** PRRSV, CD163, purification, compounds

## Abstract

**Background:**

Porcine reproductive and respiratory syndrome virus (PRRSV) has been acknowledged as one of the most important agents affecting swine. The scavenger receptor CD163 is one of the important entry mediators for PRRSV.

**Results:**

The tD4 and tD5 CD163 genes were amplified, and the PCR products were cloned into pET-28a(+) (designated pET-28a-tD4 and pET-28a-tD5, respectively). The plasmids pET-28a-tD4 and pET-28a-tD5 were then transformed into the *E. coli *BL21 (DE3) strain and expressed by adding 1 mmol/L of isopropyl-beta-D-thiogalactopyranoside. The proteins were highly expressed in the supernatant from the tD4- and tD5-producing cells that were incubated with a binding buffer containing the following compounds: β-mercaptoethanol, urea, Tween 20, glycerol, and SDS, while they were rarely expressed in the supernatant from the tD4- and tD5-producing cells that were incubated with binding buffer without the compounds. The tD4 and tD5 proteins were purified, and BALB/c mice were immunized with the purified proteins. Western blotting analysis showed that the tD4 and tD5 proteins were capable of reacting with tD5 antibodies; the titer of both the tD4 and tD5 antiserums was 1:160 against the tD5 protein, as shown by ELISA.

**Conclusions:**

These studies provide a new way for the purification of proteins expressed in inclusion bodies and the preparation of the corresponding antibodies.

## Background

Porcine reproductive and respiratory syndrome (PRRS) has been one of the most important threats to the swine industry since it was first identified in the United States in 1987[1], then in Europe in 1990[2], and later in China in 1995[3]. The clinical manifestations of PRRS are severe reproductive failure in sows, which includes early farrowing with stillborn piglets and late-term abortion, respiratory distress in piglets and growing pigs, as well as an influenza-like disease in grow-finish swine. Since 2006, a highly pathogenic PRRS virus (PRRSV), which is characterized by high fever and a high proportion of deaths in pigs of all ages, has emerged in some swine farms in China[4,5].

**Figure 1:**
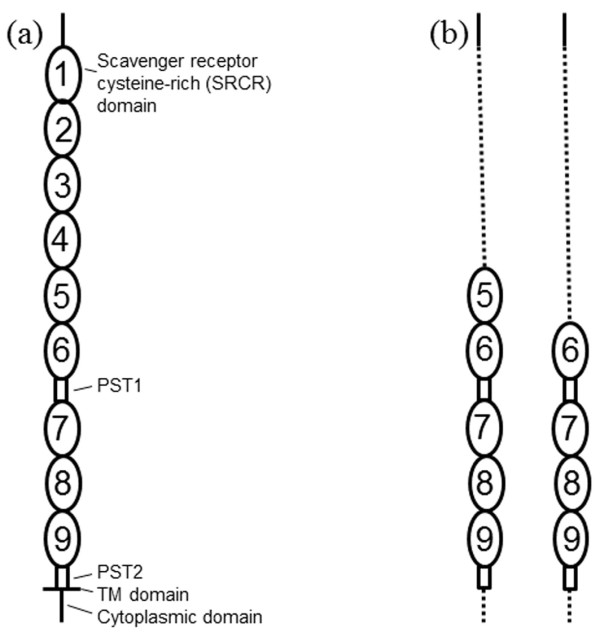
**CD163 deletion constructs were used to prepare a polyclonal antibody of the fragment of the extracellular domain**. (a) The structural domain organization of CD163 consists of 9 extracellular scavenger receptor cysteine-rich (SRCR) domains, 2 proline-serine-threonine (PST)-rich domains, a transmembrane region (TM domain), and a cytoplasmic tail. (b) The domain organization of the CD163 deletion mutants: For the tD4 mutant, the first 4 SRCR domains, the TM domain, and the cytoplasmic domain were deleted from wild type CD163. For the tD5 mutant, the first 5 SRCR domains, the TM domain, and the cytoplasmic domain were deleted from wild type CD163.

Several cellular factors involved in PRRSV binding and internalization have been studied, including sialoadhesin[6,7], heparinlike[8,9], vimentin[10], scavenger receptor CD163[11,12], and nonmuscle myosin heavy chain II-A[13]. CD163, an extracellular protein, consists of a signal peptide, 9 scavenger receptor cysteine-rich (SRCR) tandem repeats numbered 1-9, a transmembrane (TM) region, and an intracellular cytoplasmic tail (Figure 1a). In order to understand the function of SRCRs in CD163, the prokaryotic expression, purification, and antibody preparation of the fragment of the extracellular domain of the receptor CD163 were performed.

## Materials and methods

### Strains, vectors, and main reagents

In this study, we used the *E. coli *strains DH5α and BL21(DE3), the expression vector pET-28a(+), and the plasmids pcDNA3.1-CD163-D4 and pcDNA3.1-CD163-D5, which were preserved in the author's laboratory. Platinum pfx DNA polymerase was purchased from Invitrogen. Restriction enzymes, DNA markers, and isopropyl-beta-D-thiogalactopyranoside (IPTG) were purchased from TaKaRa. T4 DNA Ligase and protein molecular weight markers were purchased from Fermentas. Plasmid Mini Kits and Gel Extraction Kits were purchased from OMEGA. Ni Sepharos 6 Fast Flow was purchased from GE Healthcare.

### PCR amplification of the CD163 tD4 and CD163 tD5 genes

Based on the CD163 sequence, the primers for the amplification of the CD163 tD4 and CD163 tD5 genes were designed using the biological software Oligo v. 6.0 and synthesized by Invitrogen (Figure 1). The forward primer was 5'-TATGAAGCTTgcATGAGCAAACTCAGAATGGTG-3' and the reverse primer was 5'-TGTACTCGAGTGTGGCTTTTTGTGGGG-3', and these primers contained the *Hin*d III and *Xho *I restriction sites (underlined), respectively.

Using the plasmids pcDNA3.1-CD163-D4 and pcDNA3.1-CD163-D5 as the templates, PCR reactions (100 μL/tube) were performed using 10 μL of 10× pfx buffer, 8 μL of dNTP mix (10 mM), 2 μL of MgSO_4 _(50 mM), 2 μL of Platinum pfx DNA polymerase, 2 μL of each primer (10 μM), 1 μL of DNA template, and 73 μL of ultrapure water. The conditions of the PCR amplification were initial denaturation at 94°C for 3 min, followed by 30 consecutive cycles of denaturation at 94°C for 30 s, annealing at 55°C for 30 s, and extension at 68°C for 105 s, and then a final extension at 68°C for 7 min. The amplified products were analyzed by electrophoresis on a 1% (w/v) agarose gel.

### Construction of the expression plasmids pET-28a-tD4 and pET-28a-tD5

The PCR products of the CD163 tD4 and CD163 tD5 genes were digested by *Hin*d III and *Xho *I and directionally ligated into the previously *Hin*d III/*Xho *I-digested expression vector, pET-28a(+). The ligation mixture was transformed into competent *E. coli *DH5α cells for storage. The positive colony was identified by restriction analysis and sequencing analysis. The extracted positive plasmids were transformed into the competent *E. coli *strain BL21(DE3).

### Protein expression, purification, and polyclonal antibody production

The pET-28a-tD4 and pET-28a-tD5 positive cloning strains were each inoculated into 5 mL of LB/Kan liquid medium and cultivated overnight. The 50 μL cultures were inoculated with 5 mL of LB/Kan for activation. When the bacterium reached the logarithmic phase (at OD_600 _of 0.5-0.6), IPTG (final concentration 1.0 mmol/L) was added in order to induce the expression of the tD4 and tD5 proteins. The level of protein expression was analyzed by SDS-PAGE. The uninduced and vector control cultures were analyzed in parallel. In order to increase the production of the recombinant proteins, the expression conditions, including the duration of induction, the concentrations of IPTG, and the composition of the binding buffer (Formula of binding buffer with compounds: 20 mM Na_3_PO_4_, 0.5 M NaCl, 20 mM imidazole, 0.5% β-mercaptoethanol, 1.3 M urea, 0.5% Tween 20, 3% glycerol, 1% SDS, pH 7.4; Formula of binding buffer without compounds: 20 mM Na_3_PO_4_, 0.5 M NaCl, 20 mM imidazole, pH 7.4.) were optimized.

The tD4 and tD5 proteins were purified by Ni Sepharos 6 Fast Flow. The samples from the Ni-column were assessed by SDS-PAGE. The purified proteins were used to immunize the BALB/c mouse to the raised antibody. The antiserum was collected by tail bleeding and stored at -70°C.

### Western blot analysis of the purification of the tD4 and tD5 antigens

Western blot was used to evaluate the protein expression of tD4 and tD5, as previously described[14]. The purification samples were subjected to SDS-PAGE with a 10% gel and electrotransferred to a nitrocellulose membrane. Nonspecific antibody-binding sites were blocked with 5% skimmed milk in PBS overnight at 4°C. The membranes were incubated with a 1:50 dilution of mouse antiserum to the tD5 protein at 37°C for 1 h and then washed 4 times with PBST (5 min each). The blot was probed with a 1:5000 dilution of Odyssey infrared (IR)-labeled secondary antibody (LI-COR) for 1 h in the dark at 37°C. Then, the membrane was washed 5 times with PBST and then twice with PBS. The blot was analyzed using the Odyssey Infrared Imaging System (LI-COR).

### Indirect ELISA for tD4 and tD5-specific antibody responses

The tD4 and tD5-specific antibody responses were determined using an indirect ELISA, with purified recombinant tD5 protein as the antigen. The 96-well ELISA plates were coated overnight at 4°C with 15 μg of recombinant tD5 protein diluted in 1,997 μL of 50 mM sodium carbonate buffer (pH 9.6). The plates were washed 3 times with PBST wash buffer (0.05% Tween-20 in PBS) and blocked for 1 h at 37°C with blocking buffer (3% BSA in PBST). After 3 washes, the serum samples were diluted by 1:20, 1:40, 1:80, 1:160, 1:320, or 1:640 in blocking buffer, added to each well (100 μL per well), and incubated for 1 h at 37°C. After 3 washes, 100 μL of HRP-conjugated goat anti-mouse IgG diluted to 1:500 in blocking buffer was added to each well, and the plates were incubated at 37°C for 1 h. After 3 washes, 50 μL of tetramethylbenzidine substrate solution was added to each well for 10 min at room temperature in the dark. The reaction was stopped by the addition of 50 μL of 1 M HCl to each well. The absorbance was read at 450 nm by using an ELISA reader.

## Results

### Gene amplification and construction of expression plasmids

**Figure 2:**
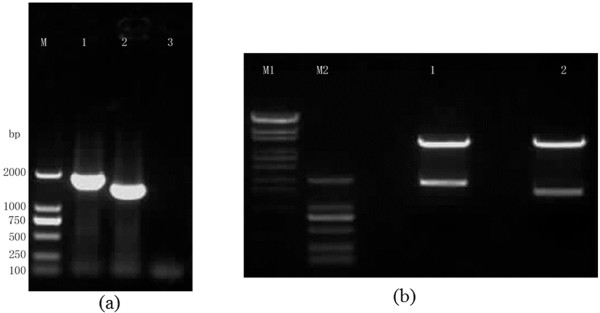
**PCR amplification of the tD4 and tD5 genes of CD163 and the restricted enzymatic digestion of pET-28a-tD4 and pET-28a-tD5**. (a) PCR amplification of the tD4 and tD5 genes of CD163: Lane M: DL2000 DNA Markers; lane 1: tD4 PCR product (1742 bp); lane 2: tD5 PCR product (1427 bp); lane 3: negative control PCR product. (b) Restriction enzyme digestion of the pET-28a-tD4 and pET-28a-tD5 plasmids: Lane M1: λ-EcoT14 I digest DNA Markers; lane M2: DL2000 DNA Markers; lane 1: Enzyme digestion of pET-28a-tD4 (5354 bp + 1728 bp); lane 2: Enzyme digestion of pET-28a-tD5 (5354 bp + 1413 bp).

Using the pcDNA3.1-CD163-D4 and pcDNA3.1-CD163-D5 plasmids as templates, the CD163 tD4 and CD163 tD5 genes were amplified. The electrophoretic analysis results of the amplified products showed that the size of the CD163 tD4 and CD163 tD5 genes were the same as expected (Figure 2a). The PCR products of the CD163 tD4 and CD163 tD5 genes were digested by *Hin*d III and *Xho *I, respectively, and directionally inserted into the pET-28a(+) plasmid in order to construct the expression plasmids. The restriction digestion analysis showed that the pET-28a-tD4 and pET-28a-tD5 expression plasmids were successfully constructed (Figure 2b).

### Protein expression and purification

**Figure 3:**
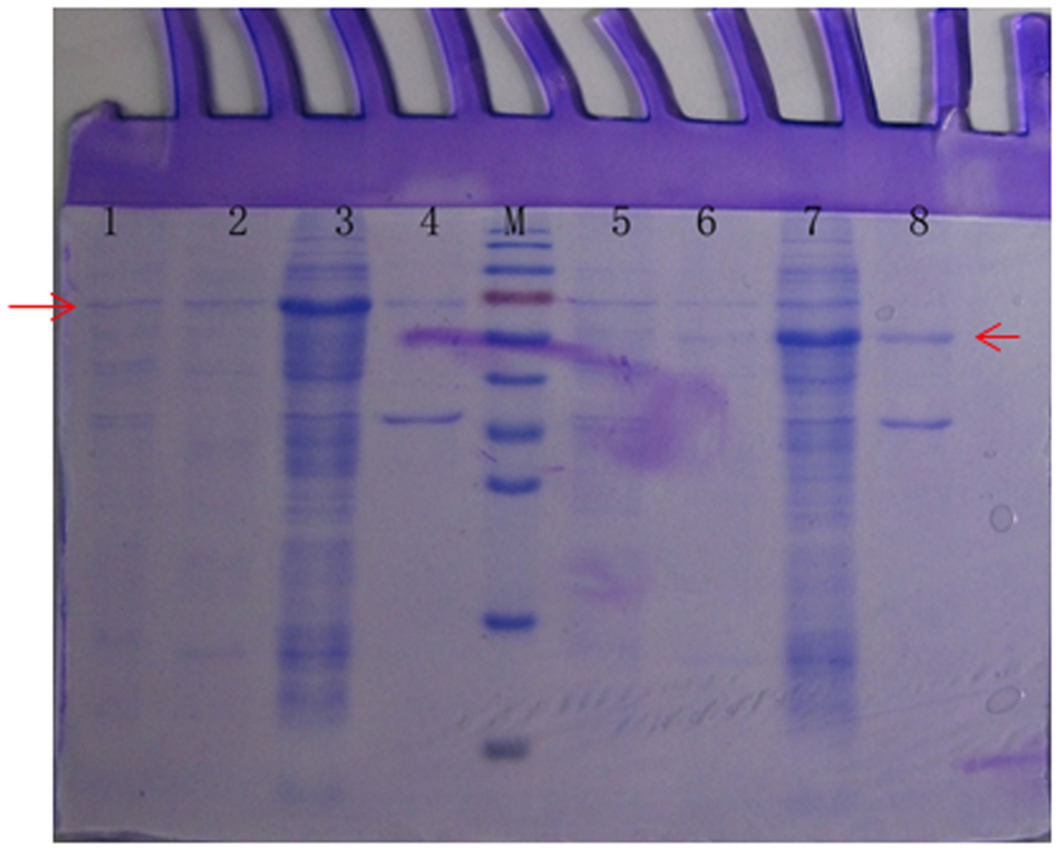
**SDS-PAGE analysis of supernatant or inclusion bodies after ultrasonic disruption**. Lane M: Protein Markers; lane 1: Supernatant after ultrasonic disruption of the tD4-producing cells that used binding buffer without the compounds; lane 2: Precipitation after ultrasonic disruption of the tD4-producing cells that used binding buffer without the compounds; lane 3: Supernatant after ultrasonic disruption of the tD4-producing cells that used binding buffer containing the compounds β-mercaptoethanol, urea, Tween 20, glycerol, and SDS; lane 4: Precipitation after ultrasonic disruption of the tD4-producing cells that used binding buffer with the compounds; lane 5: Supernatant after ultrasonic disruption of the tD5-producing cells that used binding buffer without the compounds; lane 6: Precipitation after ultrasonic disruption of the tD5-producing cells that used binding buffer without the compounds; lane 7: Supernatant after ultrasonic disruption of the tD5-producing cells that used binding buffer with the compounds; lane 8: Precipitation after ultrasonic disruption of the tD5-producing cells that used binding buffer with the compounds.

The levels of protein expression were analyzed by SDS-PAGE. The optimized conditions for the expression of the recombinant tD4 and tD5 proteins were induced 4 h after the addition of 1.0 mmol/L IPTG (Additional file 1: Fig. S1 and Additional file 2: Fig. S2); the proteins were highly expressed in the supernatant from the tD4- and tD5-producing cells that were incubated with a binding buffer containing the following compounds: β-mercaptoethanol, urea, Tween 20, glycerol, and SDS, while they were rarely expressed in the supernatant from the tD4- and tD5-producing cells that were incubated with binding buffer without the compounds (Figure 3). The recombinant tD4 and tD5 proteins were purified from the induced bacterial cells by using Ni Sepharose 6 Fast Flow gravity-flow columns (Additional file 3: Fig. S3).

### Polyclonal antibody production and western blot analysis and indirect ELISA

**Figure 4:**
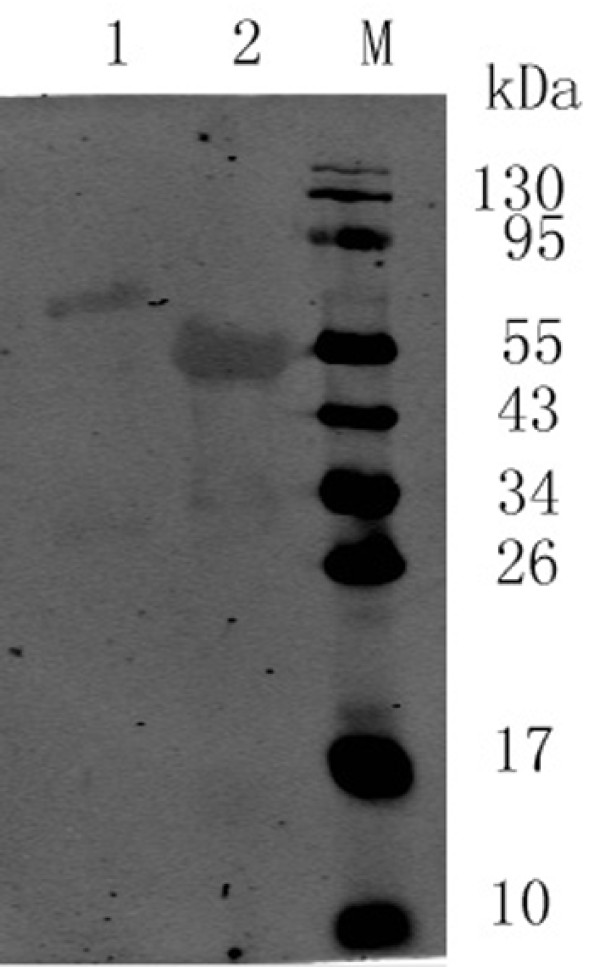
**Western blot analysis of tD4 or tD5 protein**. Lane M: Protein Markers; lane 1: tD4 protein; lane 2: tD5 protein.

The proteins were used to immunize BALB/c mice. After 3 injections, the mice anti-tD4 or anti-tD5 serum was collected by tail bleeding and stored at -70°C. In order to evaluate the level of protein expression of tD4 and tD5, western blot was used with the anti-tD5 serum as the antibody. The results showed that the proteins were reactive to the anti-tD5 serum (Figure 4). The tD4 and tD5-specific antibody responses were determined using an indirect ELISA, with purified recombinant tD5 protein as the antigen, and the ELISA results showed that the titer of both of the antibodies was 1:160.

## Discussion

PRRSV is the causative agent of PRRS and is characterized by severe reproductive failure in sows, including early farrowing of stillborn piglets and late-term abortions, respiratory distress in piglets and growing pigs, as well as an influenza-like disease in grow-finish swine. PRRS is one of the most economically important diseases affecting the swine industry worldwide. A highly pathogenic PRRSV emerged in some swine farms in China in 2006, and the infection was characterized by high fever and a high proportion of deaths in pigs of all ages[4,5]. Although modified live attenuated vaccines and inactivated vaccines against PRRSV have been available for more than a decade, the disease remains difficult to control[15,16].

PRRSV infects fully differentiated cells of the monocyte/macrophage lineage. CD163 was shown to be a cellular receptor capable of mediating infection of otherwise PRRSV non-permissive cell lines. A previous report showed that sialoadhesin and CD163 join forces during entry of the PRRSV[11]. In addition, SRCR5 is essential for PRRSV infection[17]. The minor envelope glycoproteins GP2a and GP4 of the PRRSV interact with the receptor CD163[18]. In order to understand the function of SRCRs in CD163 and their potential interplay with other receptors, the prokaryotic expression, purification, and antibody preparation of the fragment of the extracellular domain of the receptor CD163 were performed.

There was an interesting phenomenon in our experiment: the recombinant tD4 and tD5 proteins were rarely expressed in the supernatant when the tD4- and tD5-producing cells used the binding buffer without the following compounds: β-mercaptoethanol, urea, Tween 20, glycerol, and SDS (Figure 3 lane 1-2 and lane 5-6), while they were highly expressed in the supernatant when the tD4- and tD5-producing cells used the binding buffer with those compounds (Figure 3 lane 3-4 and lane 7-8). Therefore, it may be beneficial for the release of the target protein if β-mercaptoethanol, urea, Tween 20, glycerol, and SDS are added to the binding buffer with which the cells are incubated. In addition, the soluble protein levels appeared to increase when SDS was added alone to the binding buffer. However, the protein expressed in the supernatant was not significantly changed when the reagents of β-mercaptoethanol, urea, Tween 20, or glycerol were added alone to the binding buffer (Additional file 4: Fig. S4). On the contrary, when 4 of the 5 compounds were added to the binding buffer, the results showed that the proteins were highly expressed even in the supernatant, when comparing the binding buffers with the 5 compounds and that with the 5 compounds with the exception of urea or Tween 20 or glycerol (Additional file 5: Fig. S5). Furthermore, similar trials were performed with 2 GST recombinant proteins, and their results were similar with His recombinant proteins (Additional file 6: Fig. S6). Though the expression levels of the recombinant proteins in the supernatant were increased in the binding buffer containing the compounds, the purification of the GST-fusion protein failed.

Using the proteins with β-mercaptoethanol, urea, Tween 20, glycerol, and SDS to immunize the BALB/c mice, the serum was collected by tail bleeding. The ELISA results showed that the titer of both antibodies was 1:160. This indicates that the 5 compounds had no significant effects on the antibody preparation. Of course, antigen-presenting may be somewhat affected by the 5 compounds, according to the hypodermic mass of the mouse. In summary, these studies lay a foundation for further study on the function of the potential role of CD163 in PRRSV entry in macrophages and its potential interplay with other receptors and provide a new way to obtain purification of the proteins expressed in inclusion bodies and the corresponding antibody preparation.

## Conclusions

In conclusions, the tD4 and tD5 proteins and their antiserums were produced successfully. Western blotting analysis showed that the tD4 and tD5 proteins were capable of reacting with tD5 antibodies; the titer of both the tD4 and tD5 antiserums was 1:160 against the tD5 protein, as shown by ELISA. These studies provide a new way for the purification of proteins expressed in inclusion bodies and the preparation of the corresponding antibodies.

## Abbreviations

bp: base pair; cDNA: complementary DNA; SRCR: scavenger receptor cysteine-rich; IPTG: isopropyl-beta-D-thiogalactopyranoside; PRRS: Porcine reproductive and respiratory syndrome; PRRSV: Porcine reproductive and respiratory syndrome virus; PBS: Phosphate Buffer Solution; PBST: Phosphate Buffered Saline Tween-20; BSA: bovine serum albumin.

## Competing interests

The authors declare that they have no competing interests.

## Authors' contributions

ZXC, FRZ and GHZ participated in the design and carried out the majority of the experiments in the study and drafted the manuscript. KJ, WWS, MFY, SHG, PRJ, WBQ and GHZ helped to carry out the experiments and draft the manuscript. All authors read and approved the final manuscript.

## Supplementary Material

Additional file 1 **Figure S1: SDS-PAGE analysis of the expression of tD4 (a) or tD5 (b) products at different times**. Lane M: Protein Markers; lane 1: pET28a non-induced; lane 2: pET28a induced for 4 h (1.0 mmol/L); lane 3: pET-28a-tD4 or pET-28a-tD5 non-induced; lane 4: pET-28a-tD4 or pET-28a-tD5 induced for 1 h (1.0 mmol/L); lane 5: pET-28a-tD4 or pET-28a-tD5 induced for 2 h (1.0 mmol/L); lane 6: pET-28a-tD4 or pET-28a-tD5 induced for 3 h (1.0 mmol/L); lane 7: pET-28a-tD4 or pET-28a-tD5 induced for 4 h (1.0 mmol/L); lane 8: pET-28a-tD4 or pET-28a-tD5 induced for 5 h (1.0 mmol/L); lane 9: pET-28a-tD4 or pET-28a-tD5 induced for 6 h (1.0 mmol/L). Click here for file

Additional file 2 **Figure S2: SDS-PAGE analysis of the expression of tD4 (a) or tD5 (b) products with different concentrations of isopropyl beta-D-thiogalactopyranoside (IPTG)**. Lane M: Protein Markers; lane 1: pET28a non-induced; lane 2: pET28a induced for 5 h (1.0 mmol/L); lane 3: pET-28a-tD4 or pET-28a-tD5 non-induced; lane 4: pET-28a-tD4 or pET-28a-tD5 induced for 5 h (0.2 mmol/L); lane 5: pET-28a-tD4 or pET-28a-tD5 induced for 5 h (0.4 mmol/L); lane 6: pET-28a-tD4 or pET-28a-tD5 induced for 5 h (0.6 mmol/L); lane 7: pET-28a-tD4 or pET-28a-tD5 induced for 5 h (0.8 mmol/L); lane 8: pET-28a-tD4 or pET-28a-tD5 induced for 5 h (1.0 mmol/L); lane 9: pET-28a-tD4 or pET-28a-tD5 induced for 5 h (1.2 mmol/L). Click here for file

Additional files 3 **Figure S3: SDS-PAGE analysis of samples taken during the purification of tD4 (a) or tD5 (b) protein**. Lane M: Protein Markers; lane 1: Supernatant after ultrasonic disruption; lane 2: Precipitation after ultrasonic disruption; lane 3: Collected flow-though during loading of tD4 or tD5 protein; lanes 4-6: Collected flow-though from washing the gravity-flow columns with binding buffer; lanes 7-8: Collected flow-though from washing the gravity-flow columns with elution buffer. Click here for file

Additional file 4 **Figure S4: SDS-PAGE analysis of supernatant or inclusion bodies (one compound was added alone to binding buffer)**. Lane M: Protein Markers; Supernatant (lane 1) or Precipitation (lane 2) after ultrasonic disruption of the tD5-producing cells that used binding buffer without the compounds; Supernatant (lane 3) or Precipitation (lane 4) after ultrasonic disruption of the tD5-producing cells that used binding buffer only with SDS; Supernatant (lane 5) or Precipitation (lane 6) after ultrasonic disruption of the tD5-producing cells that used binding buffer only with glycerol; Supernatant (lane 7) or Precipitation (lane 8) after ultrasonic disruption of the tD5-producing cells that used binding buffer only with β-mercaptoethanol; Supernatant (lane 9) or Precipitation (lane 10) after ultrasonic disruption of the tD5-producing cells that used binding buffer only with Tween 20; Supernatant (lane 11) or Precipitation (lane 12) after ultrasonic disruption of the tD5-producing cells that used binding buffer only with urea; Supernatant (lane 13) or Precipitation (lane 14) after ultrasonic disruption of the tD5-producing cells that used binding buffer containing the five compounds: β-mercaptoethanol, urea, Tween 20, glycerol, and SDS. Click here for file

Additional file 5 **Figure S5: SDS-PAGE analysis of supernatant or inclusion bodies (four compounds were added to binding buffer)**. Lane M: Protein Markers; Supernatant (lane 1) or Precipitation (lane 2) after ultrasonic disruption of the tD5-producing cells that used binding buffer with the five compounds: β-mercaptoethanol, urea, Tween 20, glycerol, and SDS; Supernatant (lane 3) or Precipitation (lane 4) after ultrasonic disruption of the tD5-producing cells that used binding buffer with the 5 compounds with the exception of urea; Supernatant (lane 5) or Precipitation (lane 6) after ultrasonic disruption of the tD5-producing cells that used binding buffer with the 5 compounds with the exception of Tween 20; Supernatant (lane 7) or Precipitation (lane 8) after ultrasonic disruption of the tD5-producing cells that used binding buffer with the 5 compounds with the exception of β-mercaptoethanol; Supernatant (lane 9) or Precipitation (lane 10) after ultrasonic disruption of the tD5-producing cells that used binding buffer with the 5 compounds with the exception of glycerol; Supernatant (lane 11) or Precipitation (lane 12) after ultrasonic disruption of the tD5-producing cells that used binding buffer with the 5 compounds with the exception of SDS; Supernatant (lane 13) or Precipitation (lane 14) after ultrasonic disruption of the tD5-producing cells that used binding buffer without the compounds. Click here for file

Additional file 6 **Figure S6: SDS-PAGE analysis of supernatant or inclusion bodies after ultrasonic disruption of the cells from the production of GST recombinant fusion protein**. Lane M: Protein Markers; lane 1: Supernatant after ultrasonic disruption of the GST-A-producing cells that used the binding buffer without the compounds; lane 2: Precipitation after ultrasonic disruption of the GST-A-producing cells that used the binding buffer without the compounds; lane 3: Supernatant after ultrasonic disruption of the cells from the production of GST-A using binding buffer with the following compounds: β-mercaptoethanol, urea, Tween 20, glycerol, and SDS; lane 4: Precipitation after ultrasonic disruption of the GST-A-producing cells that used binding buffer with the compounds; lane 5: Supernatant after ultrasonic disruption of the GST-B-producing cells that used the binding buffer without the compounds; lane 6: Precipitation after ultrasonic disruption of the GST-B-producing cells that used the binding buffer without the compounds; lane 7: Supernatant after ultrasonic disruption of the GST-B-producing cells that used the binding buffer with the compounds; lane 8: Precipitation after ultrasonic disruption of the GST-B-producing cells that used the binding buffer with the compounds. Click here for file
